# Editorial: New therapeutic approaches in microsatellite stable colorectal cancer patients

**DOI:** 10.3389/fonc.2023.1240963

**Published:** 2023-07-21

**Authors:** Michela Bartolini, Andreas Seeber, Alberto Puccini

**Affiliations:** ^1^ Scientific Institute for Research, Hospitalization and Healthcare (Istituto di Ricovero e Cura a Carattere Scientifico) (IRCCS) Humanitas Research Hospital, Humanitas Cancer Center, Medical Oncology and Haematology Unit, Rozzano, Milan, Italy; ^2^ Department of Biomedical Sciences, Humanitas University, Pieve Emanuele, Milan, Italy; ^3^ Department of Hematology and Oncology, Comprehensive Cancer Center Innsbruck, Medical University of Innsbruck, Innsbruck, Austria

**Keywords:** MSS, colorectal cancer, immunotherapy, anti-EGFR inhibitors, liquid biopsy

Colorectal cancer (CRC) is the third most frequent cancer type diagnosed globally and the second cause of cancer-related deaths (1). The limited availability of treatment options is one of the main reasons explaining the high mortality rate among patients with advanced CRC. This is why developing effective and tolerable therapies is paramount. Fluoropyrimidine-based chemotherapy in combination with anti-VEGF and/or anti-EGFR agents is deemed the main strategy for the treatment of advanced CRC, but a better selection of patients becomes necessary to improve outcomes and avoid invariable resistance to therapy. As of today, the only biomarkers tied to clear clinical implications are *KRAS/NRAS* and *BRAF* mutations, as well as microsatellite instable/mismatch repair (MSI/MMR) status. Nevertheless, emerging biomarkers such as HER2 amplification or neurotrophic tyrosine receptor kinase (NTRK) fusions are promising in that they led to new molecularly based treatment options (2, 3). MMR-deficient (dMMR) tumors have proved notable responses to immune checkpoint inhibitor (ICI) therapy (4). However, the vast majority of patients (about 95%) are microsatellite stable (MSS) and do not benefit from immunotherapy (5).

Doleschal et al. analyzed the main resistance mechanisms to anti-EGFR monoclonal antibodies (moAbs) and deeply evaluated the well-known concepts of EGFR targeting in metastatic CRC (mCRC) in light of new diagnostic tools. They focused on liquid biopsy as a clinical practice, which has gained momentum as a powerful tool to find newly emerged mutations and as a main driver for anti-EGFR rechallenge. More specifically, differently from continued EGFR blockade, rechallenging *RAS* wild-type mCRC tumors with EGFR inhibitors in the third or later line after adequate EGFR inhibitor-free intervals appears a worthwhile strategy, which gained wider clinical applicability using liquid biopsy as explored in different studies reported in the review (Doleschal et al.). Consistent with this line of research, the increasing availability of next-generation sequencing panels (NGS) and liquid biopsy in the clinical environment allowed Mauri et al. to identify, in a real-world setting, the potential role of *MAP2K1 K57N* mutation as a negative predictive factor of response and mechanisms of primary resistance to anti-EGFR moAbs (Mauri et al.).

As far as the role of immunotherapy is concerned, the introduction of immune checkpoint inhibitors has overturned treatment and outcomes in patients with MSI mCRC. There is, however, increasing evidence that some chemotherapeutics are able to increase the immunogenicity of MSS/pMMR tumors, restoring immunotherapy sensitivity through different biological mechanisms (6). Moreover, preclinical studies have demonstrated that anti-EGFR moAbs such as cetuximab may also modulate the immune response (7). In this Research Topic, two studies explored the potential use of chemotherapy and cetuximab, in combination with ICI, for their activity in sensitizing cells to immunotherapy.

Tintelnot et al. developed the AVETUX trial, a single-arm trial that combined first-line mFOLFOX6 and avelumab with cetuximab in *RAS/BRAF* wild-type mCRC patients. The primary endpoint of 12 months PFS rate was not reached, but a strong median depth of response of 67.5% tumor shrinkage and deepness of response were observed. Moreover, translational analysis was conducted and tumor-infiltrating lymphocyte diversity and clonality, as well as peripheral blood mononuclear cell diversity after three cycles of chemotherapy, were found to be potential markers for treatment response to ICI chemotherapy combinations in mCRC. If validated in a larger cohort, these findings may be used to stratify patients or design clinical trials to increase the number of patients benefitting from ICI in MSS mCRC (Tintelnot et al.).

Along the same line of research, Napolitano et al. proposed a phase II CAVE-2 trial to evaluate the efficacy of the combination of an anti-PD-L1 IgG1 moAb avelumab plus cetuximab as a rechallenge strategy in pre-treated *RAS/BRAF* wild-type mCRC patients, treated in first line with chemotherapy in combination with cetuximab. The study aims to demonstrate a benefit in OS from treatment with cetuximab plus avelumab vs cetuximab in monotherapy, suggesting the potential synergism between immune checkpoint inhibitors and anti-EGFR drugs (Napolitano et al.).

Two reviews of the literature included in this Research Topic aimed at taking stock of our knowledge of immune-resistance mechanisms in MSS and available therapeutic strategies to overcome them.

Both reviews reported the main reasons for the lack of response to immunotherapy in MSS mCRC, such as low levels of tumor-infiltrating lymphocytes and low tumor-related neoantigens. Since CRCs with a proficient mismatch-repair pathway do not accumulate mutations, tumor molecular burden is low and tumor microenvironment is not pro-inflammatory, defining them as “cold” tumors. In addition, the reviews overviewed several trials investigating immunotherapy-based combination strategies. As emerged from both analyses, to date, none of the combinations using immunotherapy showed improvements in clinical outcomes (Ros et al.; Gandini et al.). However, Gandini et al. report recent evidence regarding the central role that intestinal microbiome could play. Microbiota could contribute not only to preventing or enhancing the risk of CRC but also to modulating the efficacy of immunotherapy (Gandini et al.).

In summary, the studies published in this Research Topic enhance our understanding of the use of anti-EGFR and the implementation of immunotherapy in MSS mCRC. This is consistent with the broad objective of expanding our knowledge of the biology underlying MSS tumors, which might ultimately lead to improved clinical trial design and to the identification of clinical biomarkers relevant to this population. In addition, the role of liquid biopsy in clinical practice may optimize the integration of EGFR inhibitors in the treatment of mCRC. The efforts of our community to improve the clinical outcomes of patients with MSS mCRC shall focus on promising available strategies, among which we certainly have better-designed drugs, including bispecific antibodies, antibody-drug conjugates (ADCs), and vaccines.

There has been increasing interest in ADCs across hematologic malignancies and solid tumors and numerous ADCs have also been evaluated in patients with advanced CRC. Future research should investigate novel payloads to which CRC is sensitive, including immune stimulating payloads, and should reconsider targeting antigens for which earlier generation ADCs failed (8). Furthermore, promising research regarding *KRAS G12C* mutation is ongoing. As reported by Doleschal et al., in a KRYSTAL-1 trial adding cetuximab to adagrasib (antiKRAS G12C inhibitor) more than doubled response rates, demonstrating how a negative feedback loop mechanism increasing EGFR signaling appeared to be responsible for treatment-related resistance; a similar mechanism was found to *BRAF V600E* inhibition (9). Lastly, another strategy worthy of clinical investigation concerns a distinct subgroup of MSS/pMMR CRC such as tumors with homologous recombination deficiency. Targeting the homologous recombination system and ICIs in this subgroup might be a potential approach that deserves further efforts (10). In conclusion, future research and clinical investigations on new combinations of treatments as well as the incorporation of new biomarkers in clinical practice become essential to further improve therapeutic strategies in MSS mCRC.

## Author contributions

MB and AP contributed to the manuscript's concept and revision. AP supervised the final version. All authors approved the submitted version.
